# Multivariate Analysis of Functional Metagenomes

**DOI:** 10.3389/fgene.2013.00041

**Published:** 2013-04-02

**Authors:** Elizabeth A. Dinsdale, Robert A. Edwards, Barbara A. Bailey, Imre Tuba, Sajia Akhter, Katelyn McNair, Robert Schmieder, Naneh Apkarian, Michelle Creek, Eric Guan, Mayra Hernandez, Katherine Isaacs, Chris Peterson, Todd Regh, Vadim Ponomarenko

**Affiliations:** ^1^Department of Biology, San Diego State UniversitySan Diego, CA, USA; ^2^Department of Computer Science, San Diego State UniversitySan Diego, CA, USA; ^3^Mathematics and Computer Science Division, Argonne National LaboratoryArgonne, IL, USA; ^4^Department of Mathematics and Statistics, San Diego State UniversitySan Diego, CA, USA; ^5^Department of Mathematics and Statistics, San Diego State UniversityCalexico, CA, USA; ^6^Computational Science Research Center, San Diego State UniversitySan Diego, CA, USA; ^7^Pomona CollegeClaremont, CA, USA; ^8^Chapman UniversityOrange, CA, USA; ^9^Torrey Pines High SchoolSan Diego, CA, USA; ^10^San José State UniversitySan José, CA, USA; ^11^Southern Oregon UniversityAshland, OR, USA

**Keywords:** metagenomics, statistics, microbiology, random forest, canonical discriminant analysis, principal component analysis

## Abstract

Metagenomics is a primary tool for the description of microbial and viral communities. The sheer magnitude of the data generated in each metagenome makes identifying key differences in the function and taxonomy between communities difficult to elucidate. Here we discuss the application of seven different data mining and statistical analyses by comparing and contrasting the metabolic functions of 212 microbial metagenomes within and between 10 environments. Not all approaches are appropriate for all questions, and researchers should decide which approach addresses their questions. This work demonstrated the use of each approach: for example, random forests provided a robust and enlightening description of both the clustering of metagenomes and the metabolic processes that were important in separating microbial communities from different environments. All analyses identified that the presence of phage genes within the microbial community was a predictor of whether the microbial community was host-associated or free-living. Several analyses identified the subtle differences that occur with environments, such as those seen in different regions of the marine environment.

## Introduction

Vast communities of microbes occupy every environment, consuming and producing compounds that shape the local geochemistry. Over the last several years sequence based approaches have been developed for the large-scale analysis of microbial communities. This technique, typically called metagenomics, involves extracting and sequencing the DNA *en masse*, and then using high performance computational analysis to associate function to each sequence. Annotation of a metagenome is conducted by comparing the sample DNA to that available in various databases, such as NCBI, SEED, MG-RAST, or COG (Wooley et al., 2010). The number of sequences similar to each protein is identified; therefore a metagenome provides information on the taxonomic make up and metabolic potential of a microbial community.

Most of the focus in metagenomics has been on single environments such as coral atolls (Wegley et al., 2007; Dinsdale et al., 2008b), cow intestines (Brulc et al., 2009), ocean water (Angly et al., 2006), and microbialites (Breitbart et al., 2009). Early work compared extremely different environments, like soil microbes compared to water microbes (Tringe et al., 2005). More recently, the Human Microbiome Project has expanded our understanding of the microbes inhabiting our own bodies, comparing samples from the same site among and between individuals (Kurokawa et al., 2007; Turnbaugh et al., 2007, 2009). These studies reflect the dynamic and expanding field of metagenomics which has been reviewed elsewhere (Wooley et al., 2010). Previously, we demonstrated that analysis of functional diversity in metagenomes could differentiate the microbial processes occurring in multiple environments (Dinsdale et al., 2008a). That study utilized the only publicly available metagenomes at that time: 45 microbial samples and 42 viral samples. The raw DNA sequences were compared to the SEED subsystems (Overbeek et al., 2005), and the normalized proportion of sequences in each subsystem in each metagenome were used as the input. That provided a raw data set with 23 response variables and 87 observations (45 microbial metagenomes and 42 viral metagenomes) or samples. In that first study, a canonical discriminant analysis (CDA) was used on a low number of samples from highly disparate environments. In this analysis, we describe a wider range of statistical analyses and use a larger sample size, to describe the abilities of metagenomes to describe the metabolic profile of microbial communities.

Even though metagenomics provides a complete analysis of the microbial activity, the results are complicated to interpret because a typical output is a list of BLAST matches to many thousands of proteins. Some programs for testing significance levels between metagenomes have been written and most use bootstrapping to avoid problems associated with the low number of replicates (Rodriguez-Brito et al., 2006; Parks and Beiko, 2010). Web based sites are being created which enable researchers to conduct statistical analysis, with no explanation of the suitability of the analysis (Arndt et al., 2012). The most common question biologists pose when conducting a metagenomic analysis is how the microbial community taxa or metabolic potential vary between sampling locations or time points. To answer this question requires the analysis and visualization of large amounts of multivariate data. To date, a few statistical tests are routinely used, including principal component analysis (PCA), multidimensional scaling (MDS), and CDA, similar to more traditional analyses of microbial communities and genomic data where PCA dominates the analyses (Ramette, 2007).

There are many statistical tools that can be used to explore multivariate data as provided by metagenomes. Here we provide an overview of seven different statistical techniques, out of the many that could be used, to compare and contrast metagenomes from different environments. In particular, we focus on tools for the classification and visualization of metagenomic data. In this work, we are concerned with how metabolic potential of the microbial community varies within and between environments.

It is important to realize that the statistical tests used will depend on the question the researcher is exploring. Not every statistical test should be used for every analysis, but several analyses can be used in combination to answer the same research question. For example, random forests are a robust analysis, but do not provide a good visualization of the data. Therefore, we combine random forest analysis with either MDS or CDA to visualize the outcome of the random forest. In this work, we have focused on clustering and visualization to show how metagenomes vary between and within environments and identify the metabolic processes that are important in driving the separations. A detailed analysis of the relationship between multivariate analyses can be found in Ramette (2007). Here we take a metagenomes centric view and briefly introduce each statistical method, and describe its ability to separate metagenomes across environmental space.

The analysis recapitulated the discriminating power of metagenomics to identify differences in functional potential both between and within environments. A unique metabolic signature represented each environmental microbial community: for example, the abundance of phage proteins was the major discriminator between host-associated microbial environments and free-living microbes. Subtle differences between open and coastal marine environments were associated with differences in the abundance of photosynthetic proteins. Cofactors, vitamins, and stress related proteins were consistently found in higher abundance in environments where the conditions for microbial survival were potentially unstable, such as hydrothermal springs. Each of these differences provides a clue for detailed microbiological analysis of communities.

## Materials and Methods

**Figure 1:**
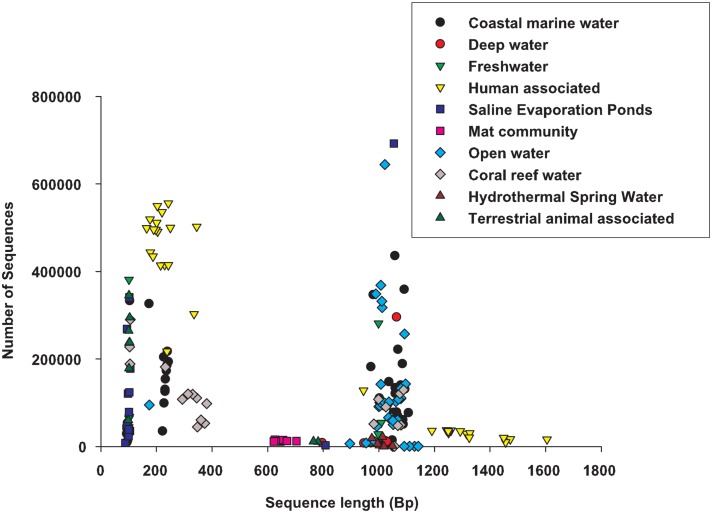
**A comparison of the sequence length and number of sequences across the environmental groups**.

At the time of analysis, 212 metagenomes were selected from the set of publicly available data^1^. They were classified into 10 different environments depending on the description provided by the researcher that collected the samples (Table A1 in Appendix). The metagenomes spanned a range of sequencing technologies, and most environments were represented by two or more sequencing technologies (Figure 1). The sample descriptions were provided as a geographical coordinate or a verbal description (e.g., coral reef water), these were translated into the environmental ontology, EnvO (Smith et al., 2006). EnvO environments were: saline evaporation pond; mat community; hydrothermal springs; human associated; other terrestrial animal associated; freshwater; and marine. Because of the abundance of samples from saline hydrographic features from the ocean (for example, Global Ocean Survey data), these samples were further sub-divided into four groups: open ocean, coastal water, deep water, and coral-reef water associated samples. The descriptions of metagenomes were mostly a geographic location, which would place the sample in a clear habitat type; a description of host, e.g., human or animal type; or a verbal description of the habitat, e.g., hydrothermal springs. There is an unfortunate lack of auxiliary data, e.g., measurements of salinity, pH, temperature, that could be used to separate the samples along a gradient. As more environmental measurements are collected at the time of metagenome sampling, the two data types (environmental and genomic) can be analyzed simultaneously to provide direct evidence of how microbial communities differ across environmental gradients and some of the statistics that we present will useful for these analysis.

Publicly available metagenomes were selected from the Edwards Lab metagenome database (see text footnote 1) (Table A1 in Appendix). All samples were annotated using the real-time *k*-mers based annotation system using a 10-amino acid word size and a requirement for at least two words per protein^2^. Real-time metagenomics: uses signature *k*-mers to identify the functions encoded in the metagenome sample (Edwards et al., 2012). The *k*-mers based approach allows all of the samples to be annotated against the same core database, and for the annotations to be updated whenever required. The *k*-mers based annotation provides the number of sequences for each function, subsystem, and two level hierarchies in the subsystems ontology (Henry et al., 2011). This system works by comparing the DNA to previously annotated DNA housed in a range of databases which identifies a gene or subsystem that shows similarity. The gene is then grouped with other genes that contribute to a metabolic pathway. The pathways are grouped with pathways that are associated with similar metabolic functions to make the top hieratical metabolic function. For example, a sequence may be similar to Alanine racemase, which is used in Alanine Biosynthesis, which is one of the pathways in Amino acid metabolism; therefore in this case the microbial community would have a sequence in the Amino acid metabolism subsystem. The counts for each metabolic process are totaled and normalized by the total number of sequences that show similarity to any subsystem. Therefore the analyses used the percent of sequences in each metabolic or functional group as the data; the metabolic group is the response variable and the metagenomes as the observations. The 27 functional hierarchies used in the analysis were: Amino Acids and Derivatives; Carbohydrates; Cell Division and Cell Cycle; Cell Wall and Capsule; Cofactors, Vitamins, Prosthetic Groups, and Pigments; DNA Metabolism; Dormancy and Sporulation; Fatty Acids, Lipids, and Isoprenoids; Membrane Transport; Metabolism of Aromatic Compounds; Miscellaneous; Motility and Chemotaxis; Nitrogen Metabolism; Nucleosides and Nucleotides; Phages, Prophages, and Transposable Elements; Phosphorus Metabolism; Photosynthesis; Plasmids; Potassium Metabolism; Protein Metabolism; Regulation and Cell Signaling; Respiration; RNA Metabolism; Secondary Metabolism; Stress Response; Sulfur Metabolism; Virulence (Aziz et al., 2008).

**Figure 2:**
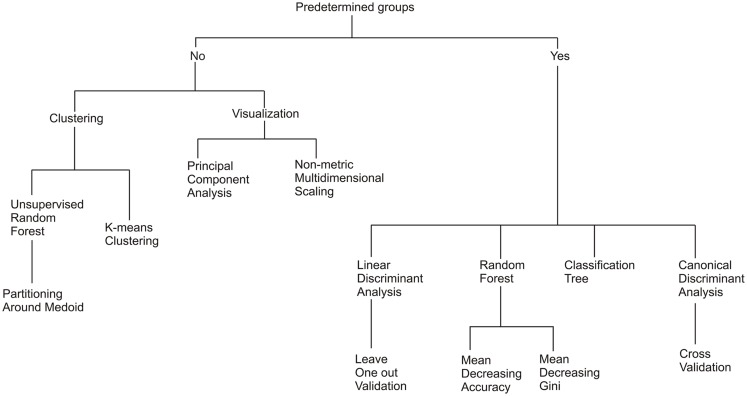
**A diagram of the relationship between the seven statistical methods evaluated**.

Common statistical techniques were used to explore the relationship between the metagenomes, environments, and subsystems (Figure 2). The two key questions addressed were: (i) do metagenomes have a metabolic signature for each environment and (ii) what are the important metabolic processes driving that signature? Clustering analysis is useful for grouping objects into categories based on their dissimilarities and work well when there is discontinuities in the samples, i.e., they are collected from distinct environments, rather than where continuous differences are expected, i.e., they are collected along a single environmental gradient. In general, statistical methods can be divided into two broad categories: supervised techniques and unsupervised techniques. Supervised techniques require that the samples be separated into predetermined groups before the analysis begins, and those groups are used as part of the analytical methods. In this case, the metagenome samples were grouped according to the environment where the sample was collected. In contrast, unsupervised techniques do not require *a priori* knowledge of the group separations, but the groups are generated by the statistical technique. In the all cases, we compare the resultant groups to the original sampled environment to determine the discriminating power of the analysis.

When categorizing data, many statistical methods are prone to over-fitting the data – reading more into the data than is really there. To reduce the problem of over-fitting the size of the data sets should be increased, groups should be of similar size and the number of groups should be less that the number of variables. Sample size considerations are particularly relevant to metagenomic data analysis, due to the nature of the data. There are thousands of proteins identified in each metagenome, but at the time of analysis there were <300 publicly available samples, meaning that there were many less samples than potential variables. Combining the proteins into functional groupings reduces the number of variables to be less than the number of samples available (subsystems were used here, but other groups like COGs, KOGs, or PFAMs are also widely used for metagenome analysis (Reyes et al., 2010). The subsystem approach is standardized and identifies all the proteins that are within a metabolic group. We used BLAST to identify how many sequences are similar to each protein. The data consisted of 10 classifications (the environments), 27 response variables (the functional metabolic groups), and 212 observations (the metagenomes). As the number of publicly available metagenomes increases the number of metabolic groups could be increased. We compared the outcome of the seven statistical analysis with the detailed methods are discussed below, and further discussion and source code for all of these operations are provided in the online accompanying material^3^. A brief summary of each method is given in the results.

### *K*-means clustering

*K*-means clustering is an unsupervised method which aims to classify observations into *K* groups, for a choice of *K*. This approach partitions observations into clusters in order to minimize the sum of squared distances from each observation to the mean of its assigned group. The function that is minimized is called the objective function describe in Eq. 1:

(1) $$\text{obj}(\mu_{1},\ldots ,\mu_{k})=\sum_{i=1}^{n}{\;}_{\mu_{1},\ldots ,\mu_{k}}^{\min}\left\|x^{\left(i\right)}-\mu_{k}\right\|$$

where *x*^(*i*)^ is an observation, μ_1_, …, μ*_k_* are the means, and *k* is such that $∥x^{\left(i\right)}-\mu_{k}∥$ is minimal. The result is *K* clusters where each observation belongs to the cluster with the closest mean.

The *K*-means algorithm starts by randomly selecting μ_1_, …, μ*_k_* and placing all observations into groups based on minimizing the objective function using Euclidean distance. The group means are then recalculated using the observations in each cluster and replace the previous means, μ_1_, …, μ*_k_*. The algorithm is repeated until additional runs no longer modify the group means or the partitioning of observations.

An alternative method of choosing *K*, uses silhouettes (Marden, 2008), which test how well an observation fits into the cluster it has been partitioned into rather than the next nearest cluster. Silhouettes give a good indication of how spread out groups are from each other. Let $a\left(i\right)=∥x^{\left(i\right)}-\mu_{k}∥\;\text{and}\;b\left(i\right)=∥x^{\left(i\right)}-\mu_{l}∥$ where *x*^(*i*)^ is an observation in group *k* and *l* is the group with the next closest mean (Marden, 2008). A silhouette is then defined in Eq. 2:

(2) $$\text{silhouette}\left(i\right)=\frac{a\left(i\right)-b\left(i\right)}{\max\left\{a\left(i\right),\;b\left(i\right)\;\right\}}$$

Ideally, each observation is much closer to the mean of its group than to the mean of any other group. In this case, the silhouette would be close to 1. Similar to the sum of squares plot, one must be careful about choosing a minimal *K* which has a large average silhouette width, though silhouette graphs frequently suggest a clear *K* to select.

### Cross-validation of classification tree

To cross validate a tree, the data set is divided into *k* randomly selected groups of near equal size. A large tree is built using the data points in only *k* − 1 groups and pruned to give a sequence of subtrees. The tree and subtrees are used to predict the classes of the remaining data points, and these predictions are compared against the actual classes of those data points. The misclassification rate and the cross-validated deviance estimate are computed for each tree, and the process is repeated for each group. This *k-*fold cross-validation procedure (Shi and Horvath, 2006) is typically repeated many times, so that different subsets are selected in each trial. The misclassification and deviance values for each tree size are averaged over there petitions, and the subtree that minimized the standard error in the misclassification rate or the lowest average deviance is selected. Trees constructed using cross-validation tools are typically less susceptible to over-fitting than other forms of classification. *K*-fold cross-validation is particularly appropriate for metagenomic data where there may be few samples in some of the environmental groups and as many samples as possible should be used to identify the right tree.

### Supervised random forest out of bagging description

Sampling the data with replacement generates a new dataset to grow each tree in the forest – a process called *bagging* (*bootstrap aggregating*). The metagenomes that are chosen at least once during the sampling process are considered *in-bag* for the resulting tree, while the remaining metagenomes are considered *out-of-bag* (OOB). Upon mature growth of the forest, each metagenome will be OOB for a subset of the trees: that subset is used to predict the class of the metagenome. If the predicted class does not match the original given class, the OOB error is increased. A low OOB error means the forest is a strong predictor of the environments that the metagenomes come from. Misclassifications contributing to the OOB errors are displayed in a *confusion matrix*. The rows in the confusion matrix represent the classes of the metagenomes and the columns represent the classes predicted by the subsets of the trees for which each metagenome was OOB. Each class error, weighted for class size, contributes to the single OOB error. The OOB error and a confusion matrix are used to judge the misclassification error and clarify where the errors occur, while the variable importance measure allows for identifying which variables are best at discriminating among groups.

### Mean decreasing accuracy and gini in supervised random forest

There are several approaches that work in conjunction with random forests to estimates the importance of variables in separating the data into groups. One uses the mean decrease in accuracy that a variable causes is determined during the OOB error calculation phase. The values of a particular variable are randomly permuted among the set of OOB metagenomes. Then the OOB error is computed again. The more the accuracy of the random forest decreases due to the permutation of values of this variable, the more important the variable is deemed.

The mean decrease in Gini is a measure of how a variable contributes to the homogeneity of nodes and leaves in the Random Forest. Let $p_{mg_{i}}$ be the proportion of samples of group *g_i_* in node *m*. Let *g_c_* be the most plural group in node *m*. The Gini index of node *mG_m_* is defined in Eq. 3:

(3) $$G_{m}=1-\underset{i\in g}{\sum }{p^{2}}_{mg_{i}}$$

The Gini index is a measure of the purity of the node, with smaller values indicating a purer node and thus a lesser likelihood of misclassification (Brieiman et al., 1984). Tree generating algorithms may use this index as their likelihood to pick which variable to split on. Each time a particular variable is used to split a node, the Gini indexes for the child nodes are calculated and compared to that of the original node. When node *m* is split into *m_r_* and *m_l_*, there is a probability $p_{m_{r}}$ of samples going into the child node *m_r_* and $p_{m_{l}}$ of going into *m_l_*. The decrease (Brieiman et al., 1984) in Gini is defined inEq. 4:

(4) $$D_{m}=G_{m}-p_{m_{r}}G_{m_{r}}-p_{m_{l}}G_{m_{l}}$$

The calculated decrease is added to the mean decrease Gini for the splitting variable and normalized at the end. The greater the mean decrease Gini of a variable, the purer the nodes splitting. Each time a particular variable is used to split a node, the Gini coefficients for the child nodes are calculated and compared to that of the original node. The Gini coefficient is a measure of homogeneity from 0 (homogenous) to 1 (heterogenous). The decreases in Gini are summed for each variable and normalized at the end of the calculation. Variables that split nodes into nodes with higher purity have a higher decrease in Gini coefficient.

### Multidimensional scaling

Multidimensional scaling is a visualization technique. Its goal is similar to PCA (see below). MDS takes for its input an *n* × *n* dissimilarity matrix *S* for *n* metagenomes, constructed by some other statistical technique, such as random forest. Then the algorithm looks for an embedding of the data points into some lower (such as 2 or 3) dimensional space that preserves the dissimilarity distances as much as possible. This embedding can then be plotted to visualize the clusters and their distances. There are various algorithms to do this, and they are rather involved. Some try to match the original distances in the embedding as well as it can. Others try to preserve the original ordering of the distances, i.e., the farther apart two samples were originally; the farther apart their images will be under the embedding.

### Linear discriminant analysis

For a data set with predetermined groups, linear discriminant analysis (LDA) constructs a classification criterion which can be used for predicting group membership of new data. LDA finds linear combinations of variables that best separate the groups, and chooses hyperplanes perpendicular to these vectors to split the data into two groups.

Let *X* be a data set with defined groups 1, …, *n*. For each group *j*, there exists a corresponding conditional distribution describe in Eq. 5.

(5) $$X\left(i\right)|G\left(i\right)=j\sim f_{j}$$

Furthermore, let π*_j_* represent the proportion of *X* that is contained in group *j*. To perform a LDA on *X*, we assume that each *f_j_* is normally distributed with an equal covariance matrix Σ, but with possibly different means μ*_j_*. Using maximum likelihood estimation theory, the linear discriminant functions can be derived in Eq. 6:

(6) $$g_{j}\left(x\right)=\log\left(\pi_{j}\right)+x\Sigma^{-1}\mu_{j}^{T}-\frac{1}{2}\mu_{j}\Sigma^{-1}\mu_{j}^{T}$$

Note that π_*j*_, μ_*j*_, and Σ are unknown parameters for our groups’ conditional distributions, so we estimate them using our sample data *X* in an intuitive manner. Suppose *X* has *N* data points and group *j* has *n_j_* points contained in it. Then we estimate π_*j*_ by ${\hat{\pi }}_{j}=\frac{n\text{\_}j}{N}$, and μ*_j_* by ${\hat{\mu }}_{j}=\sum_{i=1}^{n}\frac{X_{i}}{n_{j}}$. Let *S_j_* be the sample covariance matrix for group *j* calculated from *X*. Also ${\hat{\Sigma }}_{j}$, is taken to be 1/*n* of the pooled covariance matrix of *X*. Consequently, ${\hat{\Sigma }}_{j}={\hat{\Sigma }}_{k}$ for all *k* ∈ {1, …, *n*}. Therefore, let $\hat{\Sigma }={\hat{\Sigma }}_{1}={\hat{\Sigma }}_{2}=\ldots ={\hat{\Sigma }}_{k}$. With our population parameters estimated from our sample data *X*, the linear discriminant functions from Eq. 6 becomes described in Eq. 7:

(7) $$g_{j}(x)=\log({\hat{\pi }}_{j})+x{\hat{\sum }}^{-1}{\hat{\mu }}_{j}^{T}-\frac{1}{2}{\hat{\mu }}_{j}{\hat{\sum }}^{-1}{\hat{\mu }}_{j}^{T}$$

Note that (5) is a linear function since $\log\left({\hat{\pi }}_{j}\right)-\frac{1}{2}{\hat{\mu }}_{j}{\hat{\Sigma }}^{-1}{\hat{\mu }}_{j}^{T}$ is a constant.

These *g_j_*’s from (5) are our classifying functions. Since for a point *x* we sought to maximize π*_j_f_j_*, our *classification criterion* is

$$\text{assign }x\text{ to group }j\text{ if }g_{j}(x)>g_{k}(x)\text{ for all }k\neq j.$$

With the classification criterion, decision boundaries between groups can be found. The decision boundaries are where the discriminant functions intersect. That is, the *decision boundary between groups j and k* is {*x*:*g_j_*(*x*) = *g_k_*(*x*)}. Therefore, the linear discriminant functions split the data space into regions. Each region corresponds to a specific group and the decision boundaries separate the regions.

The original derivation of LDA (Fisher, 1936), the classifier did not start with the multivariate normal distribution. Instead, he sought the linear combination of variables that maximized the ratio of the separation of the class means to the within group variance. The pooled covariance is used in his derivation, which assumes the covariance of the groups is equal. Even though our motivation and derivation are different we still end up with Fisher’s coefficients (Venables and Ripley, 2002).

To judge how well a given LDA acts as a classifier for new data, *leave one out* cross-validation can be can be used and is implemented in the Statistical Package R (2009). Let *X* be a data set with *m* data points, and with groups 1, …, *n*. For an LDA carried out on *X*, leave one out cross-validation removes one observation, *x*^(*i*)^, at a time from *X*, performs an LDA on the reduced data set, and then uses this new LDA to classify *x*^(*i*)^. Since the group membership of *x*^(*i*)^ is already known, we can check if the quasi-LDA for *X* classifies *x*^(*i*)^ correctly or not. For every observation in *X*, the procedure of leaving one out, and classifying with a new LDA is performed. The number of *p* of misclassifications is found. The proportion *p*/*m* is an estimate for the probability of the LDA carried out on *X* misclassifying a new observation.

### Principal component analysis

Principal component analysis is a dimension reduction technique. It uses orthonormal linear combinations of the variables of the data, called principal components, to capture most of the variance in a few dimensions. The idea is to choose the first principal component so that it has maximal variance, and each successive principal component so that it absorbs as much of the remaining variance as possible. The number of principal components of a dataset is equal to the number of variables, but most of the variance is concentrated in the first few.

Given an *n* × *q* data matrix *Y* with corresponding *q* × *q* covariance matrix *S*, the *q* × 1 principal component vectors *ν*_1_,…, *ν_n_* are described in Eq. 8:

(8) $${\langle \nu_{\iota },\nu_{j}\rangle }_{\;\left\|\nu_{\iota }\right\|=1}^{{\;}_{=0,1\leq j<i}^{\max}\left\|\nu_{\iota }S\nu_{i}^{Τ}\right\|}$$

Since *S* is a symmetric matrix, the spectral theorem shows that all of its eigenvalues are real and that it has an orthonormal basis of eigenvectors (Marden, 2011). Hence it follows that the principal components of *Y* are the eigenvectors of *S* ordered by decreasing eigenvalues.

The principal components of *Y* capture all of the variance of the variables. PCA is an effective tool when the first few principal components account for most of the variance. In practice, being able to capture over 95% of the variance in the first two principal components is not unusual. Then the data can be plotted along the first two or three principal components to visualize clustering. If the first few principal components fail to account for most of the variance, it indicates that the data is inherently multidimensional.

### Canonical discriminant analysis

Canonical discriminant analysis centers on the construction of canonical components to explain the variance between classes. For a data set with variables (ν_1_, ν_2_, … , ν*_k_*), these canonical components are linear combinations of the form shown in Eqs 9 and 10:

(9) $$\begin{array}{rc} \text{Can1} & ={\hat{a}}_{1}\nu_{1}+{\hat{a}}_{2}\nu_{2}+\ldots +{\hat{a}}_{k}\nu_{k} \end{array}$$

(10) $$\begin{array}{rc} \text{Can2} & ={\hat{b}}_{1}\nu_{1}+{\hat{b}}_{2}\nu_{2}+\ldots +{\hat{b}}_{k}\nu_{k} \end{array}$$

For two-dimensional visualization it is necessary to project the variable vectors *v*_1_ and *v*_2_, onto the canonical component axes Can1 and Can2 (Marden, 2011). The projections of the variables maintain the relationship between their coefficient variables. That is shown in Eq. 11:

(11) $$\frac{a_{i}}{b_{i}}=\frac{{\hat{a}}_{i}}{{\hat{b}}_{i}}\;\text{and}\;\frac{a_{i}}{b_{j}}=\frac{{\hat{a}}_{i}}{{\hat{b}}_{j}}$$

The amount of the inter-class variance that is explained by each component is indicated in parentheses along each axis. The vectors can be rescaled to obtain the clearest visualization, but they must maintain the ratio of their lengths as this is proportional to their importance. Each sample is plotted according to its canonical scores. Let *x* be a sample, such that *x* = (*x*_1_, *x*_2_, …, *x_k_*) from a data set whose first canonical components are 𝒞_1_ and 𝒞_2_, such that the coefficients of 𝒞_1_ are (*a*_1_, *a*_2_, …, *a_k_*) and those of 𝒞_2_ are (*b*_1_, *b*_2_, …, *b_k_*). Then we compute using Eq. 12:

(12) $$\begin{array}{llll} x𝒞 & =x\left(\;\begin{array}{c} |\\ {𝒞}_{1}\\ | \end{array}\;\begin{array}{c} |\\ {𝒞}_{2}\\ | \end{array}\right)=\left(x_{1},\;x_{2},\;\ldots ,\;x_{k}\right)\left(\;\begin{array}{c} a_{1}\\ a_{2}\\ \vdots \\ a_{k} \end{array}\;\begin{array}{c} b_{1}\\ b_{2}\\ \vdots \\ b_{k} \end{array}\right) & & \\ & =\left({𝒞}_{1}\left(x\right)\;{𝒞}_{2}\left(x\right)\right) \end{array}$$

The canonical scores of a sample *x* are (𝒞_1_(*x*), 𝒞_2_(*x*)), which describe its position in the 2-dimensional space defined by the first two canonical components. The mean scores and confidence intervals of the means can also be plotted.

The choice of group was determined by the minimal Mahalanobis distance. The Mahalanobis measure is a scale-invariant distance measure based on correlation. The distance of a multivariate vector *x* = (*x*_1_, *x*_2_, …, *x_k_*) from a group with mean μ = (μ_1_, μ_2_, …, μ*_n_*) and covariance matrix *S* is defined in Eq. 13:

(13) $$D_{M}\left(x\right)=\sqrt{\left(x-\mu \right)S^{-1}{\left(x-\mu \right)}^{T}}$$

More intuitively, consider the ellipsoid that best represents the group’s probability density. The Mahalanobis distance is simply the distance of the sample point from the center of mass, divided by the spread (width of the ellipsoid) in the direction of the sample vector (Marden, 2011).

## Results

### Overview

We begin by assessing the clustering of the metagenomes and test whether the clusters chosen to reflect the environmental signals are statistically supported (*K*-means, decision trees, and random forests). We then move on to methods to explore and visualize the underlying structure of the data (MDS, linear discriminant, principal components, and CDA). An outline of the statistical methods tested is shown in Figure 2. Obviously statistical analysis is not a linear process, and many of the techniques were influenced by the results from previous (or subsequent) analyses. Although this discussion attempts to maintain a linear structure for readability, that is not always possible or appropriate. Often the researcher will have a specific biological question and a single specific statistical analysis will be appropriate. A combination of statistical tests can provide better visualization of the data. For example random forests are good at recognizing important variables and how the observations are divided or classified, but do not provide data visualization tools. Therefore, we used a random forest analysis to provide the clustering and a MDS plot to visualize the data.

### *K*-means clustering

The most straightforward method to cluster data is by grouping into related sets. *K*-means clustering aims to classify observations into *K* groups by partitioning observations into clusters in order to minimize the sum of squared distances from each observation to the mean of its assigned group. The *K*-means algorithm starts by randomly selecting a specified number of means and groups observations by assigning each one to the mean it is closest to in distance. The group means are then recalculated using the observations, replacing the previous means. The observations are reassigned to a group based on the distance between the value and the mean of the group. The algorithm iterates until the groups stabilize. The algorithm will converge to a local minimum, but not necessarily to a global minimum, therefore it is necessary to initialize and run the analysis many times.

**Figure 3:**
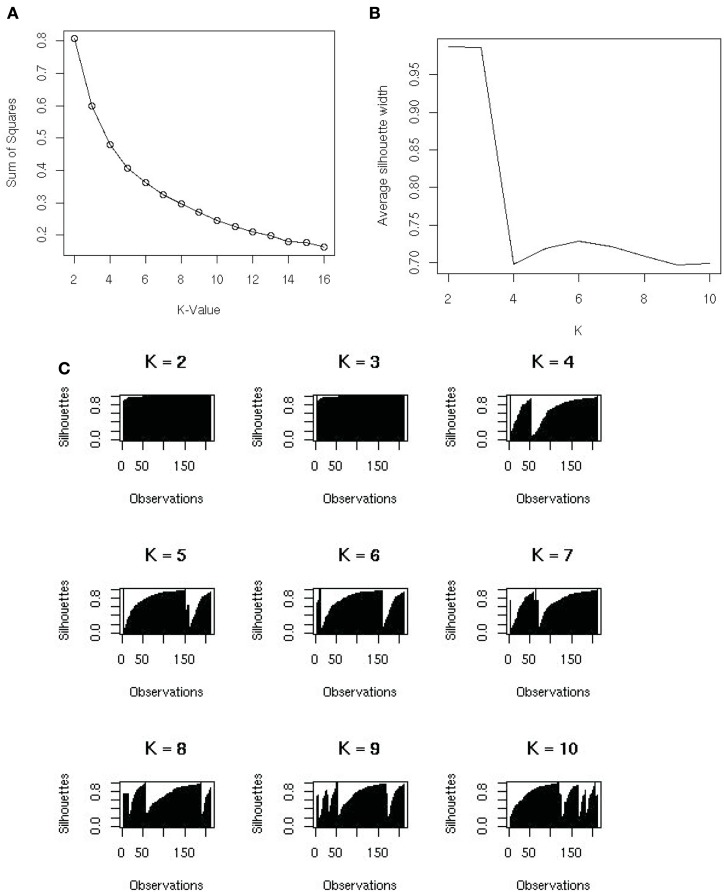
**(A)** The sums of squares and *K*-value used to identify the number of groups that the samples should be split into. No clear elbow was evident; therefore silhouette plots were used to examine the data. **(B)** A silhouette plot showing how it creates metagenomic groups in the data. The most favorable grouping number is where the average silhouette width is nearest to one. **(C)** The variation of average silhouette width and *K*. There is a peak at *K* = 6 and an uptick at *K* = 10.

Varying the number of groups (*K*) will result in different results from the *K*-means algorithm. The sum of squares of distances in general decreases as *K* increases, because there are more groups in which to assign observations. Selecting *K* with the smallest sum of squares will over-fit the data. In fact, when *K* is the number of observations, each observation will form a group by itself and the sum of squares will be 0; but this does not give any useful information about the data. A plot of the sum of squares versus values of *K* is useful for determining an optimal value of *K* (Figure 3A). *K* is often selected where the plot has an “elbow.” However, with metagenomic data, the plot often appeared rounded (Figure 3A), therefore, we optimized using silhouettes (Rousseeuw, 1987) instead. The silhouette of an observation is the difference between its distances from the closest of the *K-*means and the second closest, divided by its distance from the second closest mean. In the best possible case, the observation is close to its own mean and not very close to the second best mean, i.e., its silhouette is close to 1. The set of all silhouettes (one for each observation) for *K* from 1 to 10 is shown in Figure 3B. For each value of *K* we calculate the average silhouette width, and use *K* that optimizes the width of the silhouettes. We found a maximum at *K* = *6*, with another smaller optimal width at *K* = *10* (Figure 3C).

The *K*-means algorithm was most useful for identifying outliers, which could be checked visually and removed as required. Using *K* = *6* groups, identified two broad categories, (1) the aquatic group cluster and (2) the human, terrestrial animal associated and mat community cluster (Table 1), but the remaining four groups were small and consisted of samples that were potential outliers. The advantage of the *K*-means approach was that it showed broad patterns in the metagenomic data. If the researcher did not know how many groups were in the dataset, this analysis would be a good place to start the analysis. The disadvantage was that it does not provide any information about which metabolic processes were driving the broad scale separations in the metagenomes.

**Table 1:** **The samples present in each of the clusters identified by the *K*-means analysis with *K* = 6**. This was chosen because the silhouette analysis suggested that six clusters were the most appropriate (Figure 3). There were 33 human, 9 terrestrial animal, 10 mat community, 42 open water, 20 reef water, 60 coastal water, 5 deep water, 7 fresh water, 15 hypersaline, and 6 hot spring samples in total.

Cluster	Number of metagenomes	Original metagenome classification
1	52	31 Human
		5 Terrestrial animals
		6 Mat community
		Water samples:
		4 Open marine
		3 Coral reef
		2 Coastal marine
		1 Fresh
2	1	1 Coral reef water sample
3	1	1 Coral reef water sample
4	3	1 Human
		1 Fresh water
		1 Coral reef water
5	149	4 Mat
		4 Terrestrial animals
		1 Human
		Water samples:
		56 Coastal marine
		5 Deep marine
		15 Solar evaporation ponds
		6 Hydrothermal spring
		38 Open mainre
		13 Coral reef
		7 Freshwater
6	6	Water samples:
		2 Coastal marine
		3 Freshwater
		1 Coral reef

### Classification trees

A supervised decision tree constructs a classification tree by identifying variables and decision rules that best distinguish between predefined classes (supervised). If the response variable is continuous, instead of predefined classes, a regression tree can be constructed which predicts the average value of the response variable. Either of these trees is suitable for metagenomic data, but since we were interested in separating the data by environment we used classification trees. Trees are invariant under monotonic transformations of the response variables, because constructing a tree uses binary partitions of the data and thus most variable scaling is unnecessary (De’ath and Fabricius, 2000; De’ath, 2002). This feature is particularly important, because a mixture of data can be included in the analysis, e.g., the percent of sequences similar to a metabolic process or the pH where the metagenome was collected. Combining genomic and environmental data will be useful in future analyses.

The construction of a supervised tree minimizes the mixing of the different predefined classes within a leaf (called the node impurity). At each branching point, the algorithm chooses a single variable and a value that splits the node minimizing the impurity. (There are several ways to measure impurity, as described in the methods) In general, trees are a balance between classification strength and model complexity with the goal of maximizing prediction strength and minimizing over-fitting. Often a large tree is grown that over-fits the data, and pruning and cross-validation are used to select the most appropriate sub-tree of that original tree (Brieiman et al., 1984).

**Figure 4:**
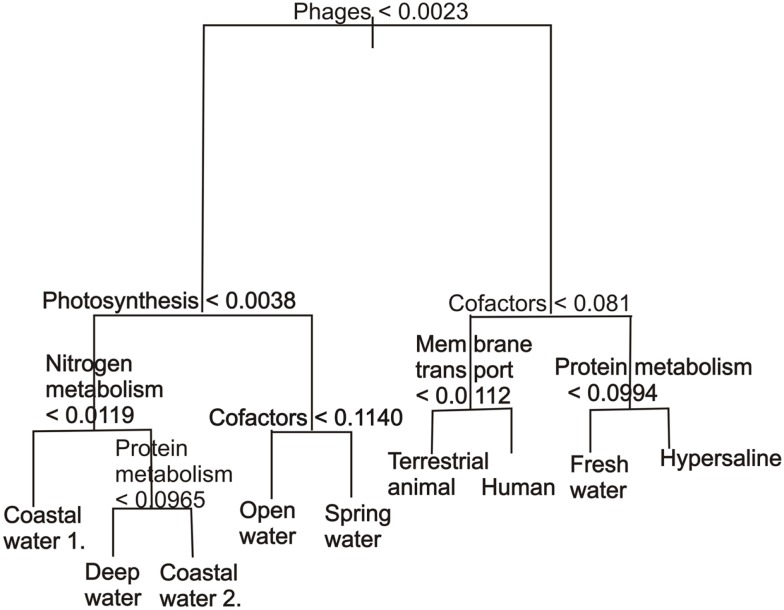
**A classification tree showing the separation of metagenomes from different environments based on the abundance of the subsystems in each environment**. The abundances are normalized as described in the methods. The tree has been pruned to only show the eight most important variables.

Unlike *K*-means clustering, decision tree classification provides information about the variables that drive the separation. The best classification tree using all the variables was determined by 500 runs of 10-fold cross-validation (Table 2). The cross-validation identified three trees that gave similarly low values, the 6, 8, and 9-leafed tree. These were visually inspected to see which tree gave information without being over-fitted and this was the 9-leafed tree. This classification tree (Figure 4) demonstrated that phage proteins separated the host-associated microbial communities and the majority of free-living communities. In particular, and as has been shown before (Oliver et al., 2009; Reyes et al., 2010), the host-associated communities and some microbial communities from the fresh water and hypersaline environments characteristically had more phage proteins.

**Table 2:** **Tree size and average deviance from a series of tree cross-validation experiments**.

Tree size	Average CV deviance
1	152.014
2	122.432
3	102.636
4	99.642
6	92.762
8	92.970
9	92.812
14	95.848
16	98.342
17	98.622

Harsh environments (such as hypersaline aquatic environments) had more cofactors, vitamins, and pigments. Within the marine realm, the coastal and deep water samples had, as expected, fewer photosynthetic proteins than the open water samples, but the photosynthetic potential of the reefs was mixed (Dinsdale et al., 2008a). Photosynthetic potential also aided the identification of stratification in the mat microbial communities by depth, a separation that was supported by metabolism that occurs in microaerobic or anoxic conditions. The major advantages of classification trees are the ability to use any continuous variable type, fast calculation time, good visualization, and the ability to calculate misclassification rate. The use of classification tree in association with environmental data in the future will be able to show the interactions between the environmental and genomic characteristics. The disadvantage is the tendency for over-fitting the trees and the lack of stability: small changes in the data, such as adding one more sample, can yield dramatically different results.

### Random forests

**Figure 5:**
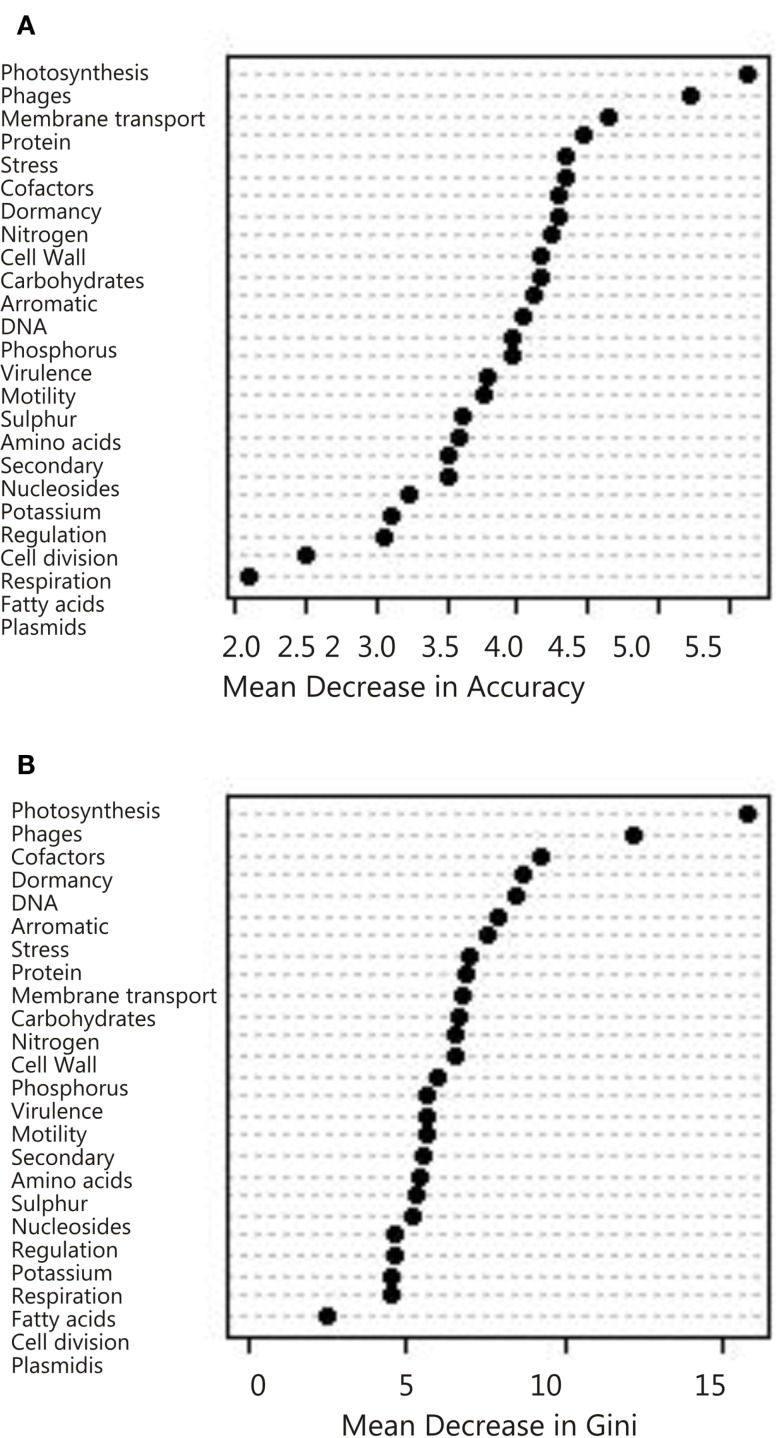
**Variable importance determined by the random forest analysis using mean decrease in (A) Accuracy and (B) Gini**.

The random forest (Brieiman, 2001) technique aims to overcome the limitations of the classification tree by generating a large ensemble of trees from a random subset of the data and a random selection of the variables. The resulting ensemble of trees (the random forest) is then used with a majority-voting approach to decide which metagenomes belong to which groups. The computation is not excessive: a random forest with 1000 trees trained on 212 metagenome datasets was computed in a few seconds. The speed of calculation and bootstrapping nature of random forests, may pave the way for calculations across all proteins in all environments, thus reducing the amount of grouping conducted on the data. The random forest is typically used to classify the data into predefined groups (a *supervised* random forest). A subset of the data and variables is used to generate the trees, and thus the approach can predict the environment to which a metagenome belongs. The random forest does not produce branching rules like a single classification tree because the trees in the random forest all differ from one another. Instead, the most parsimonious tree is calculated using bagging (Table A1 in Appendix). In addition to bagging, the RF generates a measure of the importance of each variable, calculated by either the mean decrease in accuracy or the mean decrease in the Gini (Figure 5). These two values indicate which variables contributed the most to generating strong trees and can be used in other visualization analyses such as MDS or CDA as described below.

In an unsupervised random forest, the metagenomic data is classified without *a priori* class specifications. Therefore, unsupervised random forests remove researcher bias. Synthetic classes are generated randomly and the forest of trees is grown. Similar metagenomes will end up in the same leaves of trees due to the tree branching process, and the *proximity* of two metagenomes is measured by the number of times they appear on the same leaf. The proximity is normalized so that a metagenome has proximity of one with itself and 1-proximity is a dissimilarity measure (Shi and Horvath, 2006). The strength of the clustering detected this way may be measured by a “partitioning around the medoids” (PAM) analysis (Marden, 2011). Conceptually similar to the *K*-means clustering, PAM picks *K* metagenomes called medoids, and creates clusters by assigning each metagenome to the group represented by its closest medoid. The algorithm looks for whichever *K* metagenomes minimize the sum of the distances between all metagenomes and their assigned medoids.

Overall, the photosynthesis and phage groups were the most important response variables in separating the data sets, and in the mean decreasing accuracy plot a break occurred between these two variables and the remaining variables, suggesting that just these two measures could be used to grossly classify the metagenomes (Figure 5). The next break appeared after the eighth variable. These eight variables were thus chosen for the CDA analysis described below. The misclassification rate of the random forest analysis was 31% (Table 3) and these misclassifications occurred because metagenomes from the various marine environments were mixed. The marine environment categories of open ocean, coastal waters, coral reef, and deep ocean, share many metabolic features and therefore these metagenomes were placed into categories different than their *a priori* group assignment. This suggests subtle variation in metabolic processes that are occurring in the microbial communities from each environment that should be investigated in the future.

**Table 3:** **The group that each metagenome was assigned to by the random forest analysis**.

Initial classification	Classification from the random forest								
	Mixed marine	Deep marine water	Coastal marine	Open marine	Hydrothermal spring water	Terrestrial animals	Human associated	Fresh water	Saline evaporation ponds
Freshwater	3				1	1			
Open marine	6	1	1	31					2
Hydrothermal spring water	1				5				
Coastal marine	6	1	43	8	2				
Terrestrial animal						5 cows 2 mice	3 mice 1 fish		
Human associated	1		1				32		
Mat community	4	1						4	
Deep marine water		4	1						
Coral reef water	4	1		15					
Saline evaporation ponds	4				1				9
Total	29	8	47	44	8	8	36	10	11

The advantages of the random forest are that it is a rapid classification technique that is less susceptible to over-fitting data and can be run in a bootstrap fashion. In addition, the random forest provides a measure of the importance of each variable that can be used in other analyses. These advantages of random forests mean that the metagenomes could be analyzed on the gene level, rather than the higher subsystem level. The disadvantage is that because each forest is an ensemble of trees, identifying individual classification decisions is not possible, which is why we plotted the data using a MDS.

### Multidimensional scaling

**Figure 6:**
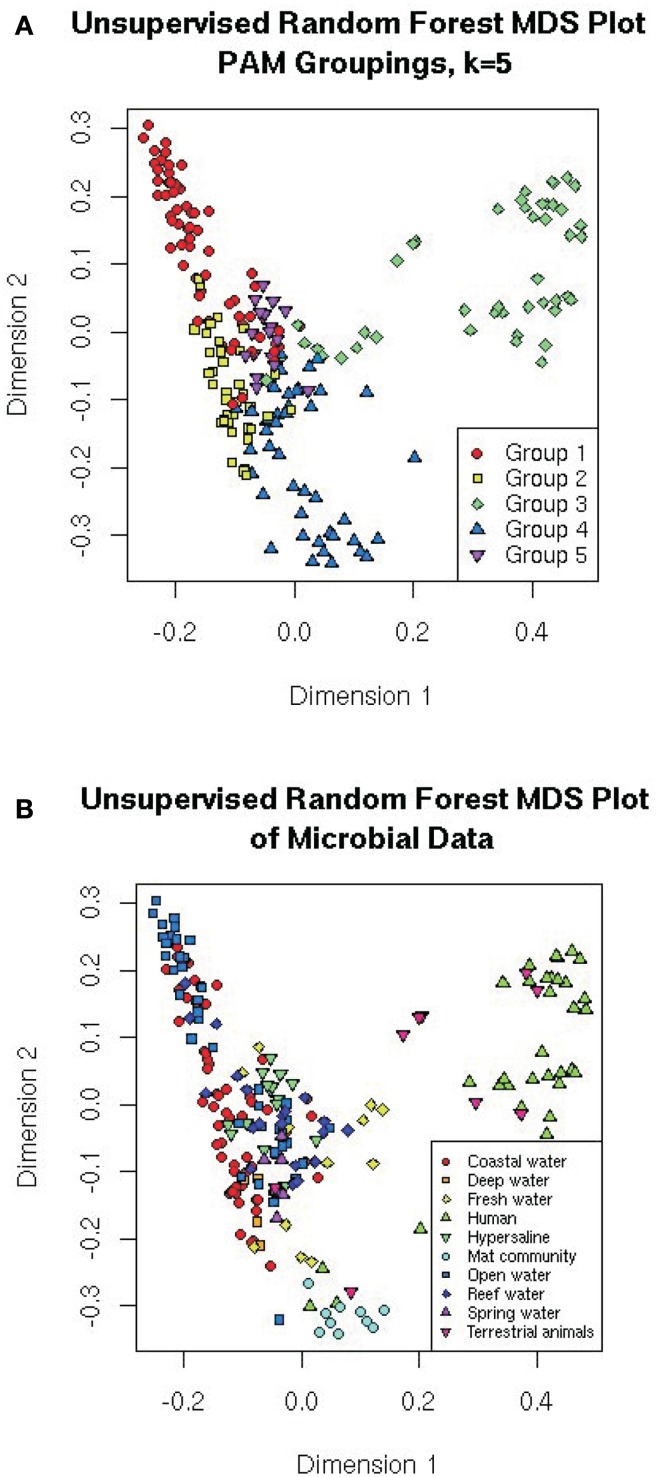
**Multiple dimensional scale plots of the distances calculated from the unsupervised random forest**. The distances are the number of times the samples appear on the same leaf of the tree, and the MDS has scaled them so that they plot projects those distances into two dimensions. Colored by **(A)** the five PAM groupings suggested by the random forest (see text); or **(B)** the original environments the samples came from.

Multidimensional scaling is a visualization tool that directly scales objects based on either similarity or dissimilarity matrices (Quinn and Keough, 2002). MDS projects the proximity measures of the metagenomes as determined by other techniques to a lower-dimensional space (e.g., 2-dimensional space for plotting on *xy*-axis). For the random forests, the similarity was measured as the number of times two metagenomes appeared on the same leaf in the trees (proximity), and is represented by the distance between two samples on the MDS plot. The MDS plots are colored either by the five PAM groupings from the random forest (Figure 6A), or the 10 predefined environments (Figure 6B). In this analysis, the visualization highlights the separation of the microbes from human/animal hosts from other samples along the first dimension and the separation of the aquatic and mat communities along the second dimension.

It is important to note that MDS is a visualization technique that takes its input from other classification or clustering approaches. MDS is useful for showing which metagenomes have similar features, because metagenomes that are positioned closer together will be more similar to each other than those farther apart on the plot.

### Linear discriminant analysis

Linear discriminant analysis is a supervised statistical technique that aims to separate the data into groups based on hyperplanes and describe the differences between groups by a linear classification criterion that identifies decision boundaries between groups.

**Figure 7:**
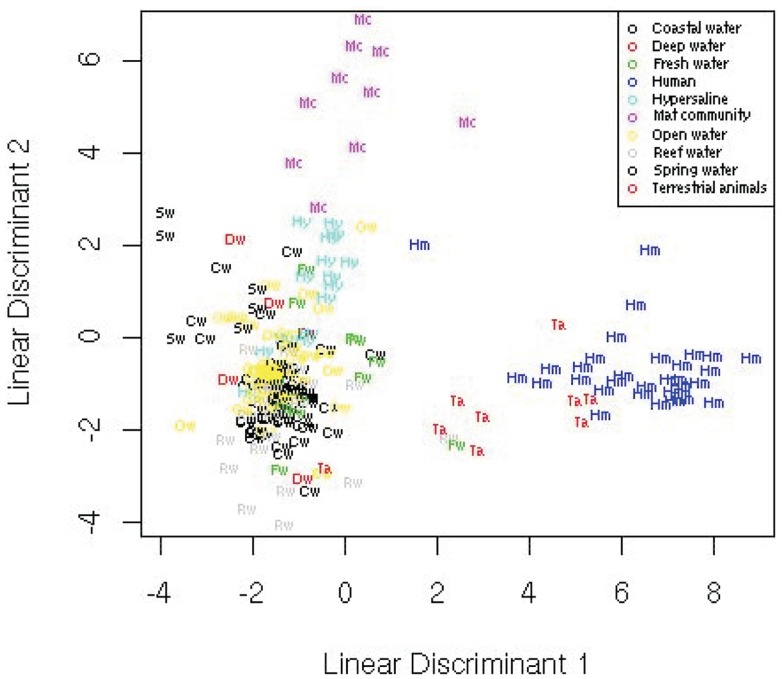
**Linear discriminant analysis showing the position of the metagenomes in two-dimensional space from the 10 environments**.

The LDA over all 27 metabolic variables separated the data (Figure 7) and showed that the human and terrestrial animal associated metagenomes separated from a cluster consisting of all of the aquatic samples except the hypersaline community. The mat samples separated distinctly from the other clusters. A leave one out cross-validation showed that the LDA misclassified 36% of the samples. Most of the misclassified samples were from the aquatic metagenomes that are difficult to separate (as discussed below). Even though it is likely that the data does not meet all the requirements for an LDA, including the assumption of equal population group covariance, a linear function of the variables is still able to separate the groups. We derive the linear discriminant functions assuming the data is normally distributed for simplicity, but this is not necessary. The advantages of LDA are the ability to both visualize the data and obtain a statistically robust classification, but the disadvantage includes the assumption of equal population covariance.

### Principal component analysis

**Figure 8:**
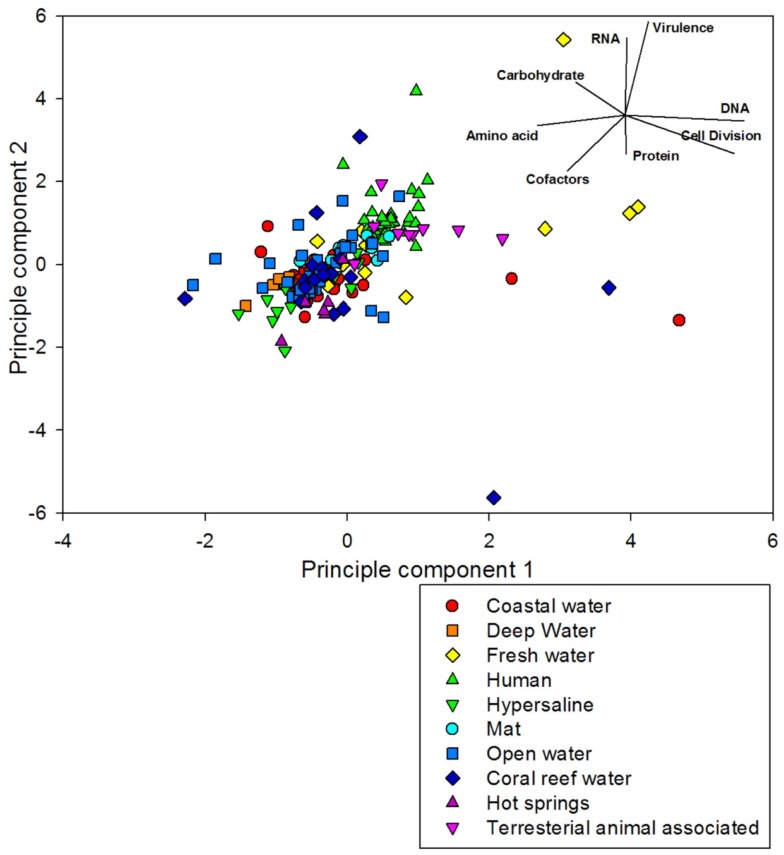
**Principal component analysis of the 212 metagenomes using the top eight variables identified from the random forest analysis**. The samples are colored and shaped by the environment where they came from. The samples are largely aligned on a 45° plane from virulence-DNA metabolism to amino acids-cofactors.

Principal component analysis is one of the most widely used statistical analyses for genomics data because it is a straightforward, robust data reduction technique that is trivial to apply to large data sets. PCA selects linear combinations of the variables sorted so that each combination accounts for as much of the sample variance as possible, while being orthogonal to the previous combinations. These combinations of the variables are called the principal components. The goal of PCA is to explain as much of the variance as possible in the first few components, and thus reduce the complexity of the data by combining related variables. We began with the eight most important variables identified by the random forest, and used PCA to reduce these to a two-dimensional plot. Figure 8 shows a PCA plot of the first two principal components of the data set, and shows the directionality of the importance of each variable.

The data was positioned on a plane which was influenced by a high percent of sequences associated with DNA metabolism, cell division, and amino acid metabolism in one direction, and virulence and RNA metabolism in the other, with cofactor metabolism important in both directions. The metagenomes did not separate particularly well with this analysis, however human and terrestrial animal associated samples clustered above aquatic samples. The first two dimensions of the PCA did not provide good resolution of the nuances within an environment, explaining only 38% of the variance. This suggests that a large number of components were needed to explain the variance in our data and highlights a problem with PCA: it is not able to reduce the complexity of the data if the variables are not correlated. The lack of correlation in the variables can be seen in Figure 8, because the metabolic processes are facing in different directions around the graph. There is no grouping of any of the 8 metabolic processes shown. We did get better resolution with PCA on certain subsets of the data for example, using some of the organism-associated metagenomes. In this case the first two principal components accounted for 79% of the variance. We did not include those graphs in this paper.

The advantages of the PCA are that it reduces the complexity of the data, especially if many of the variables are correlated, and it provides a mechanism for visualizing higher-dimensionality data. The disadvantages of the PCA are that it does not classify the metagenomes into groups and if the variables are not correlated it is unable to reduce the dimensionality of the data.

### Canonical discriminant analysis

Canonical Discriminant Analysis is another approach to reduce the dimensionality of the data, similar to PCA and LDA. However, in addition to visualizing the data, CDA can be used to classify the data into pre-assigned groups. Like the PCA, CDA searches for linear combinations of variables that explain the data. Like a supervised random forest, CDA can be used to explore the variables responsible for differentiating between groups.

**Figure 9:**
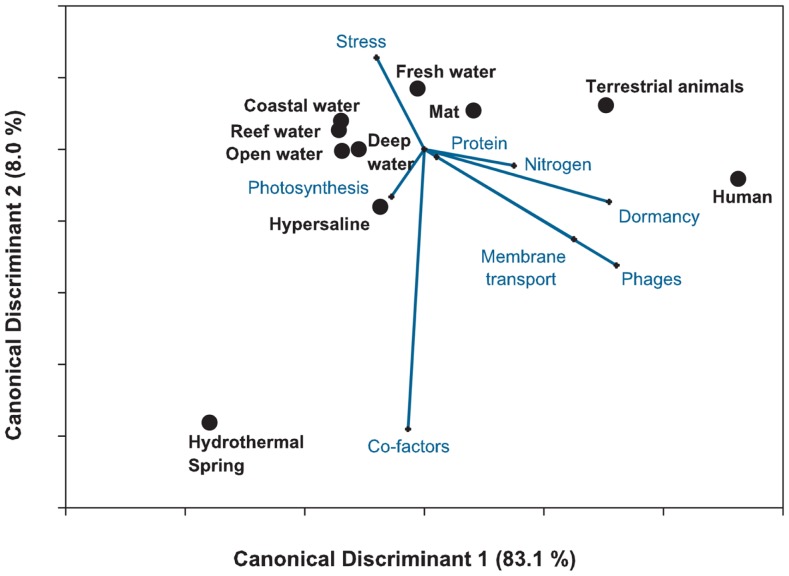
**Canonical discriminant analysis of the 212 metagenomes using the top eight variables identified from the random forest analysis**. The plot shows the separation in the host-associated microbial communities and the free-living communities. The analysis explained 91% of the variance, suggesting that metagenomes can be discriminated by the metabolic potential. Lines depict the h-plot of important metabolic processes and the points are the centroid or mean for the 10 environments.

CDA identifies variation between groups by identifying the linear combination of variables that has the maximum multiple correlations with the groups. The second component is the linear combination that has the highest possible multiple correlations within the groups and is uncorrelated with the first component. The process is repeated using all the data, and providing one fewer components than variables. A fundamental difference between PCA and CDA is the covariance matrix: in the former the covariance matrix displays the variance between individual samples, while in the latter it displays the variance between groups. As with the PCA, we explored the effect of the eight most important response variables on the separation of the 212 metagenomes using CDA (Figure 9) and found the mediods of the groups and vectors that demonstrate the directionality of the importance of each variable. The length of the vector in the plot is proportional to the importance of that variable in separating the data.

The CDA showed that the host-associated microbial communities were separated from the other environments by the abundance of sequences similar to phage and dormancy proteins. The harsh hydrothermal springs were again associated with the need for cofactors. The photosynthetic potential separated the coastal and open water metagenomes. Membrane transport, protein and nitrogen metabolism were also important in separating the aquatic from host-associated metagenomes. The analysis explained a large amount of the variance (91%) showing the importance of a key set of metabolic processes in each environment. However, the misclassification rate of the CDA was 39.7%. Once again the largest misclassification occurred between the metagenomes collected from the four marine environments (Table 4).

**Table 4:** **The misclassification table generated by the canonical discriminant analysis**.

	Coastal marine water	Deep marine water	Fresh water	Human	Solar evaporative ponds	Mat community	Open marine water	Coral reef water	Hydrothermal spring	Terrestrial animal	Class error
Coastal marine water	9.820	0.000	0.301	0.391	0.000	0.226	0.962	0.009	0.127	0.160	0.181
Deep marine water	0.990	0.004	0.000	0.000	0.000	0.000	0.004	0.000	0.000	0.000	0.995
Freshwater	0.816	0.000	0.433	0.231	0.000	0.235	0.081	0.028	0.160	0.075	0.783
Human	0.000	0.000	0.207	6.268	0.000	0.457	0.014	0.051	0.000	0.000	0.104
Solar evaporative ponds	1.231	0.000	0.000	0.000	1.485	0.000	0.283	0.000	0.000	0.000	0.504
Mat community	0.382	0.000	0.000	0.004	0.000	1.613	0.000	0.000	0.000	0.000	0.193
Open marine water	4.377	0.009	0.033	0.448	0.169	0.349	2.410	0.169	0.014	0.018	0.698
Coral reef	1.509	0.009	0.283	0.429	0.000	0.226	1.117	0.235	0.023	0.377	0.994
Hydrothermal spring	0.047	0.000	0.000	0.000	0.000	0.000	0.113	0.004	0.834	0.000	0.165
Terrestrial animal	0.287	0.000	0.108	1.193	0.000	0.216	0.000	0.000	0.000	0.193	0.903

The advantages of the CDA are that it combines the dimensionality reduction of the PCA with the classification of the random forest or *K*-means approaches. The disadvantages of the CDA are that the metagenomes are placed into predefined groups and thus are subject to observer bias, and CDA is prone to over-fitting because the canonical components are linear combinations that best separate the groups.

## Discussion

Metagenomic data provides a wealth of information about the functional potential of microbial communities, but the vastness of the data makes it difficult to discern patterns and important discriminators. A range of clustering, classification and visualizing techniques were applied to analyze metagenomic data, and demonstrated the ability of the metabolic profiles to describe the difference between environments. The results show that a mixture of methods provides an effective analysis of the data: *K*-means was used to identify outliers, random forests to identify the most important variables, and either a classification tree or CDA to test the relevance of the environment to genomic content.

The data generation processes could cause differences in the classification or separation of the data. However the samples came from multiple sources, each of which employed a range of isolation, purification, and sequencing techniques. There was no evidence of clustering of samples prepared or sequenced in a specific manner, suggesting that the sampling technique *per se* is not driving the separation of the data.

The analyses separated the microbial samples into three broad groups (based on the environments from where they were isolated): the human and animal associated samples, the microbial mats, and the aquatic samples. There was a clear difference between environments. For example, human associated and aquatic samples were clearly separated by all of the techniques. However, samples from a similar environment were often misclassified. For example, the coastal and open water metagenomes were difficult to classify. More sampling and more thorough description of the environmental parameters will clarify the classification of these samples.

The combination of random forests and CDA demonstrated that phage activity is a major separator of host-associated microbial communities and free-living or environmental microbial communities, suggesting that the phages are playing different ecological roles within each environment. In free-living microbial communities, phages are major predators and generally show similar diversity to their hosts. In host-associated microbial communities, phages are more diverse suggesting that they may provide specific genes to increase host survival (Reyes et al., 2010). The mat communities separated from both the animal associated metagenomes and the aquatic samples by the vitamin and cofactor metabolism, suggesting a role for secondary metabolism associated with growth in extreme environments. The dominant metabolism that separated the aquatic samples was photosynthesis. Not surprisingly, samples from deep in the ocean, and some of the impacted reef sites, do not have many photosynthetic genes, while photosynthetic genes abound on unaffected reefs and in surface waters of the open ocean. Although only the one or two most abundant phenotypes in each sample were described here, the statistical analysis reveals less obvious separations among the data, and unraveling the role of microbes in the global geobiology is an important goal for post-metagenomic studies.

In summary, we hope that the statistical tools described here will help microbial ecologists broaden the range of statistical tools that are used in metagenomic data and help them parse out the important and interesting nuances that separate different environments.

## Conflict of Interest Statement

The authors declare that the research was conducted in the absence of any commercial or financial relationships that could be construed as a potential conflict of interest.

## Footnotes

^1^http://edwards.sdsu.edu/mymgdb/

^2^http://edwards.sdsu.edu/rtmg

^3^http://dinsdalelab.sdsu.edu/metag.stats/

## Appendix

**Table A1:** **Metagenomes used in the analysis**.

Environment	Genome ID	Genome name	Project	Num. of sequences	Total length (bp)
Coastal water	4441143	GS009 – Coastal Block Island, NY – USA	Global ocean sampling	79,303	84,327,514
Coastal water	4441144	GS010 – Coastal Cape May, NJ – USA	Global ocean sampling	78,304	82,424,426
Coastal water	4441148	GS117b – Coastal Indian Ocean – St. Anne Island, Seychelles	Global ocean sampling	50,609	54,752,102
Coastal water	4441152	GS004 – Coastal Outside Halifax, Nova Scotia – Canada	Global ocean sampling	52,959	56,922,096
Coastal water	4441153	GS007 – Coastal Northern Gulf of Maine – Canada	Global ocean sampling	50,980	55,430,960
Coastal water	4441579	GS002 – Coastal Gulf of Maine – Canada	Global ocean sampling	121,590	128,761,768
Coastal water	4441580	GS003 – Coastal Browns Bank, Gulf of Maine – Canada	Global ocean sampling	61,605	66,907,344
Coastal water	4441581	GS005 – Embayment Bedford Basin, Nova Scotia Canada	Global ocean sampling	61,131	65,983,125
Coastal water	4441582	GS006 – Estuary – Bay of Fundy, Nova Scotia – Canada	Global ocean sampling	59,679	64,615,563
Coastal water	4441583	GS008 – Coastal Newport Harbor, RI – USA	Global ocean sampling	129,655	137,725,898
Coastal water	4441584	GS012 – Estuary Chesapeake Bay, MD – USA	Global ocean sampling	126,162	136,081,077
Coastal water	4441585	GS013 – Coastal – Off Nags Head, NC – USA	Global ocean sampling	138,033	149,007,574
Coastal water	4441586	GS015 – Coastal – Caribbean Sea – Off Key West, FL – USA	Global ocean sampling	127,362	138,034,062
Coastal water	4441589	GS019 – Coastal – Northeast of Colon – Panama	Global ocean sampling	135,325	146,413,090
Coastal water	4441591	GS021 – Coastal – Gulf of Panama – Panama	Global ocean sampling	131,798	143,454,700
Coastal water	4441595	GS027 – Coastal – Devil’s Crown, Floreana Island – Ecuador	Global ocean sampling	222,080	237,326,008
Coastal water	4441596	GS029 – Coastal – North James Bay, Santigo Island – Ecuador	Global ocean sampling	131,529	143,822,814
Coastal water	4441596	GS028 – Coastal Floreana Ecuador	Global ocean sampling	189,052	205,008,796
Coastal water	4441597	GS030 – Warm Seep Upwelling, Fernandina Island	Global ocean sampling	436,401	461,671,889
Coastal water	4441598	GS032 – Mangrove – Mangrove on Isabella Island – Ecuador	Global ocean sampling	148,018	153,341,974
Coastal water	4441600	GS034 – Coastal – North Seamore Island – Ecuador	Global ocean sampling	134,347	142,199,308
Coastal water	4441601	GS035 – Coastal – Wolf Island – Ecuador	Global ocean sampling	140,814	151,840,270
Coastal water	4441602	GS036 – Coastal – Cabo Marshall, Isabella Island – Ecuador	Global ocean sampling	77,538	85,757,477
Coastal water	4441605	GS049 – Coastal – Moorea, Outside Cooks Bay – Fr. Polynesia	Global ocean sampling	92,501	94,424,378
Coastal water	4441613	GS117a – Coastal St. Anne Island, Seychelles	Global ocean sampling	346,952	339,868,195
Coastal water	4441618	GS149 – Harbor – West coast Zanzibar Tanzania	Global ocean sampling	110,984	111,178,553
Coastal water	4441658	GS011 – Estuary Delaware Bay, NJ – USA	Global ocean sampling	124,435	133,251,132
Coastal water	4441659	GS014 – Coastal South of Charleston, SC – USA	Global ocean sampling	128,885	139,914,998
Coastal water	4441660	GS016 – Coastal Sea Gulf of Mexico – USA	Global ocean sampling	127,122	137,479,949
Coastal water	4441662	GS030 – Warm Seep – Roca Redonda – Ecuador	Global ocean sampling	359,152	391,694,924
Coastal water	4440358	DMSP21SeawaterMic200511	Marine manipulated	41,461	3,882,661
Coastal water	4440359	VAN11SeawaterMic200511	Marine manipulated	29,104	2,710,130
Coastal water	4440360	DMSP2SeawaterMic200511	Marine manipulated	50,313	4,813,851
Coastal water	4440361	VAN21SeawaterMic200511	Marine manipulated	40,480	3,867,992
Coastal water	4440362	DMSP11SeawaterMic200511	Marine manipulated	44,246	4,202,321
Coastal water	4440363	VAN2SeawaterMic200511	Marine manipulated	33,773	3,269,294
Coastal water	4440364	DMSP1SeawaterMic200511	Marine manipulated	54,848	5,279,589
Coastal water	4440365	VAN1SeawaterMic200511	Marine manipulated	12,446	1,190,841
Coastal water	4443688	BBAY01	Botany bay metagenomic	71,068	75,802,328
Coastal water	4443689	BBAY02	Botany bay metagenomic	13,512	13,814,160
Coastal water	4443691	BBAY04	Botany bay metagenomic	14,708	15,408,753
Coastal water	4443693	BBAY15	Botany bay metagenomic	182,393	177,136,646
Coastal water	4443702	SRS000294	Coastal waters plymouth	204,693	46,327,791
Coastal water	4443703	SRS000295	Coastal waters plymouth	130,806	30,141,333
Coastal water	4443704	SRS000296	Coastal waters plymouth	326,310	56,526,614
Coastal water	4443706	SRS000299	Coastal waters plymouth	154,069	35,762,224
Coastal water	4443707	SRS000298	Coastal waters plymouth	126,086	29,082,158
Coastal water	4443708	SRS000300	Coastal waters Plymouth	35,712	7,909,745
Coastal water	4443709	SRS000301	Coastal waters plymouth	99,488	22,554,197
Coastal water	4443711	SRS000536_2	Marine synechococcus experiment	333,462	34,334,174
Coastal water	4443712	mb2000jd298_2	Monterey bay microbial study	194,144	46,983,239
Coastal water	4443713	mb2000jd298_1	Monterey bay microbial study	217,549	51,966,974
Coastal water	4443714	mb2001jd115_1	Monterey bay microbial study	186,172	44,189,510
Coastal water	4443715	mb2001jd115_2	Monterey bay microbial study	173,161	40,680,713
Coastal water	4443718	SRS000238	Sapelo island metagenome	49,524	4,719,520
Coastal water	4443719	SRS000239	Sapelo island metagenome	46,421	4,361,030
Coastal water	4443720	SRS000240	Sapelo island metagenome	44,317	4,209,153
Coastal water	4443721	SRS000242	Sapelo island metagenome	9,967	933,470
Coastal water	4443722	SRS000241	Sapelo island metagenome	41,537	3,890,082
Coastal water	4443724	SRS000243	Sapelo island metagenome	30,673	2,940,585
Deep water	4441025	Mediterranean Bathypelagic Habitat	Mediterranean bathypelagic habitat	9,047	7,202,361
Deep water	4441041	HOT/ALOHA – Below Base of Euphotic Zone 200m	Hot/aloha	8,276	7,829,627
Deep water	4441056	HOT/ALOHA – Deep Abyss 4000m	Hot/aloha	11,223	11,028,802
Deep water	4441057	HOT/ALOHA – Well Below Upper Mesopelagic 500m	Hot/aloha	9,017	8,764,614
Deep water	4441062	HOT/ALOHA – Core of Dissolved Oxygen Minimum Layer 770m	Hot/aloha	11,478	11,811,596
Deep water	4441590	GS020 – Fresh Water – Panama Canal – Lake Gatun – Panama	Global ocean sampling	296,355	315,151,139
Freshwater	4443679	AntarcticaAquatic_3	Antarctica aquatic microbial	10,042	9,755,315
Freshwater	4443680	AntarcticaAquatic_2	Antarctica aquatic microbial	9,672	9,622,231
Freshwater	4443681	AntarcticaAquatic_4	Antarctica aquatic microbial	54,446	54,929,769
Freshwater	4443683	AntarcticaAquatic_1	Antarctica aquatic microbial	100,085	101,310,476
Freshwater	4443684	AntarcticaAquatic_6	Antarctica aquatic microbial	281,490	281,056,691
Freshwater	4443685	AntarcticaAquatic_7	Antarctica aquatic microbial	28,481	28,413,296
Freshwater	4443687	AntarcticaAquatic_9	Antarctica aquatic microbial	95,521	95,664,001
Freshwater	4440411	PrePondKentSTMic20060504	Freshwater from aquaculture facility	44,094	4,428,989
Freshwater	4440413	TilPondKentSTMic20060504	Freshwater from aquaculture facility	63,978	6,484,135
Freshwater	4440422	TilPondKentSTMic200608	Freshwater from aquaculture facility	67,612	6,932,903
Freshwater	4440440	TilPondKentSTMic200511	Freshwater from aquaculture facility	381,076	38,804,235
Human associated	4441092	Australian Phosphorus Removing (EBPR) Sludge	Phosphorus removing (ebpr) sludge	96,563	100,273,005
Human associated	4441093	US Phosphorus Removing (EBPR) Sludge	Phosphorus removing (ebpr) sludge	127,953	120,938,054
Human associated	4440453	TS1	Gut microbiome	217,386	51,708,794
Human associated	4440454	TS2	Twin study	443,640	78,853,892
Human associated	4440461	TS4	Twin study	414,754	95,003,113
Human associated	4440462	TS5	Twin study	490,776	100,599,979
Human associated	4440463	TS6	Twin study	535,763	118,207,161
Human associated	4440595	TS3	Twin study	510,972	102,717,417
Human associated	4440610	TS19	Twin study	498,880	82,117,565
Human associated	4440611	TS20	Twin study	495,040	98,053,098
Human associated	4440613	TS28	Twin study	302,780	101,434,082
Human associated	4440614	TS49	Twin study	519,072	91,987,878
Human associated	4440615	TS50	Twin study	549,700	111,999,603
Human associated	4440616	TS29	Twin study	502,399	173,386,030
Human associated	4440639	TS21	Twin study	413,772	88,786,017
Human associated	4440640	TS51	Twin study	434,187	81,330,211
Human associated	4440823	TS7	Twin study	555,853	134,889,015
Human associated	4440824	TS8	Twin study	414,497	100,520,072
Human associated	4440825	TS30	Twin study	495,865	94,405,318
Human associated	4440826	TS9	Twin study	499,499	124,768,172
Human associated	4440939	human F1-S	Human feces – kurokawa	28,900	38,010,851
Human associated	4440940	human F1-U	Human feces – kurokawa	16,539	24,369,492
Human associated	4440941	human F1-T	Human feces – kurokawa	36,326	43,259,070
Human associated	4440942	human F2-V	Human feces – kurokawa	36,455	45,906,118
Human associated	4440943	human F2-W	Human feces – kurokawa	30,198	40,076,128
Human associated	4440944	human F2-X	Human feces – kurokawa	31,237	39,071,077
Human associated	4440945	human In-B	Human feces – kurokawa	9,958	14,499,070
Human associated	4440946	human In-A	Human feces – kurokawa	20,226	29,296,224
Human associated	4440947	human F2-Y	Human feces – kurokawa	35,177	45,480,292
Human associated	4440948	human In-D	Human feces – kurokawa	37,296	46,397,089
Human associated	4440949	human In-M	Human feces – kurokawa	16,164	25,941,797
Human associated	4440950	human In-E	Human feces – kurokawa	20,532	27,208,886
Human associated	4440951	human In-R	Human feces – kurokawa	34,797	43,473,860
Solar evaporation ponds	4441050	Marine NaCl-Saturated Brine	Marine nacl-saturated brine	2,947	2,380,900
Solar evaporation ponds	4441599	GS033 – Hypersaline Floreana Island – Ecuador	Global ocean sampling	692,255	729,708,089
Solar evaporation ponds	4440324	LowSalternSDbayMic20051110	Solar saltern	49,074	4,632,200
Solar evaporation ponds	4440329	SaltonSeaMic20060823	Solar saltern	178,407	18,876,339
Solar evaporation ponds	4440416	MedSalterSDbayMic20051128	Solar saltern	8,062	705,995
Solar evaporation ponds	4440419	HighSalternSDbayMic20051128	Solar saltern	35,446	3,711,295
Solar evaporation ponds	4440425	MedSalternSDbayMic20051116	Solar saltern	120,987	11,867,028
Solar evaporation ponds	4440426	LowSalternSDbayMic20051128	Solar saltern	34,296	3,453,306
Solar evaporation ponds	4440429	HighSalternSDbayMicB200407	Solar saltern	39,553	4,028,912
Solar evaporation ponds	4440430	HighSalternSDbayMicA200407	Solar saltern	78,524	7,982,909
Solar evaporation ponds	4440433	HighSalternSDbayMicC200407	Solar saltern	123,879	12,641,571
Solar evaporation ponds	4440434	MedSalternSDbayMic20051111	Solar saltern	23,261	2,323,241
Solar evaporation ponds	4440435	MedSalternSDbayMic20051110	Solar saltern	38,929	3,905,955
Solar evaporation ponds	4440437	LowSalternSDbayMic200407	Solar saltern	268,206	25,280,522
Solar evaporation ponds	4440438	HighSalternSDbayMicD200407	Solar saltern	340,725	34,806,789
Mat community	4440963	Guerrero Negro 1–2 mm	Hypersaline guerro negro	11,562	7,469,278
Mat community	4440964	Guerrero Negro 0–1 mm	Hypersaline guerro negro	12,213	8,596,197
Mat community	4440965	Guerrero Negro 2–3 mm	Hypersaline guerro negro	12,407	8,286,254
Mat community	4440966	Guerrero Negro 3–4 mm	Hypersaline guerro negro	12,821	8,214,974
Mat community	4440967	Guerrero Negro 4–5 mm	Hypersaline guerro negro	15,652	9,803,688
Mat community	4440968	Guerrero Negro 10–22 mm	Hypersaline guerro negro	12,686	8,016,534
Mat community	4440969	Guerrero Negro 5–6 mm	Hypersaline guerro negro	12,525	8,376,984
Mat community	4440970	Guerrero Negro 6–10 mm	Hypersaline guerro negro	15,048	9,863,015
Mat community	4440971	Guerrero Negro 22–34 mm	Hypersaline guerro negro	12,522	8,382,531
Mat community	4440972	Guerrero Negro 34–49 mm	Hypersaline guerro negro	11,627	7,240,219
Open water	4441051	HOT/ALOHA – Upper Euphotic 10m	Hot/aloha	7,837	7,482,115
Open water	4441055	HOT/ALOHA – Base of Chlorophyll Maximum 130m	Hot/aloha	6,797	6,091,740
Open water	4441057	HOT/ALOHA – Upper Euphotic 70m	Hot/aloha	10,992	10,828,356
Open water	4441125	GS040 – Open Ocean – Tropical South Pacific	Global ocean sampling	736	772,365
Open water	4441126	GS041 – Open Ocean – Tropical South Pacific	Global ocean sampling	678	739,958
Open water	4441127	GS042 – Open Ocean – Tropical South Pacific	Global ocean sampling	699	788,466
Open water	4441128	GS043 – Open Ocean – Tropical South Pacific	Global ocean sampling	711	789,468
Open water	4441129	GS044 – Open Ocean – Tropical South Pacific	Global ocean sampling	678	714,813
Open water	4441130	GS045 – Open Ocean – Tropical South Pacific	Global ocean sampling	730	796,793
Open water	4441131	GS046 – Open Ocean – Tropical South Pacific	Global ocean sampling	626	683,240
Open water	4441134	GS110b – Open Ocean – Indian Ocean –	Global ocean sampling	49,597	53,607,277
Open water	4441135	GS120 – Open Ocean – Indian Ocean – Madagascar	Global ocean sampling	46,052	45,710,196
Open water	4441136	GS039 – Open Ocean – Tropical South Pacific	Global ocean sampling	759	866,795
Open water	4441139	GS122b – Open Ocean Madagascar and South Africa	Global ocean sampling	50,096	52,667,848
Open water	4441145	GS037 – Open Ocean – Eastern Tropical Pacific	Global ocean sampling	65,670	68,651,473
Open water	4441146	GS047 – Open Ocean – Tropical South Pacific	Global ocean sampling	66,023	68,340,256
Open water	4441147	GS112b – Open Ocean – Indian Ocean	Global ocean sampling	52,118	55,638,894
Open water	4441149	GS116 – Open Ocean – Indian Ocean	Global ocean sampling	60,932	64,223,447
Open water	4441150	GS115 – Open Ocean – Indian Ocean	Global ocean sampling	61,020	64,230,062
Open water	4441151	GS119 – Open Ocean – Indian Ocean	Global ocean sampling	60,987	65,055,874
Open water	4441155	GS109 – Open Ocean – Indian Ocean	Global ocean sampling	59,813	62,752,349
Open water	4441156	GS111 – Open Ocean – Indian Ocean	Global ocean sampling	59,080	62,072,289
Open water	4441570	GS000a – Open Ocean – Sargasso Sea	Global ocean sampling	644,551	658,755,696
Open water	4441573	GS000b – Open Ocean – Sargasso Sea	Global ocean sampling	317,180	321,026,307
Open water	4441574	GS000c – Open Ocean – Sargasso Sea	Global ocean sampling	368,835	371,688,861
Open water	4441575	GS000d – Open Ocean – Sargasso Sea	Global ocean sampling	332,240	335,939,509
Open water	4441576	GS001a – Open Ocean – Sargasso Sea	Global ocean sampling	142,352	143,316,448
Open water	4441577	GS001b – Open Ocean – Sargasso Sea	Global ocean sampling	90,901	90,951,299
Open water	4441578	GS001c – Open Ocean – Sargasso Sea	Global ocean sampling	92,351	92,688,958
Open water	4441587	GS017 – Open Ocean – Yucatan Channel – Mexico	Global ocean sampling	257,581	281,259,325
Open water	4441588	GS018 – Open Ocean – Rosario Bank – Honduras	Global ocean sampling	142,743	156,474,992
Open water	4441592	GS022 – Open Ocean – Eastern Tropical Pacific	Global ocean sampling	121,662	131,079,270
Open water	4441594	GS026 – Open Ocean – Galapagos Islands	Global ocean sampling	102,708	109,049,397
Open water	4441607	GS110a – Open Ocean – Indian Ocean	Global ocean sampling	99,288	100,097,831
Open water	4441609	GS110a – Open Ocean – Indian Ocean	Global ocean sampling	99,781	101,818,659
Open water	4441610	GS110a – Open Ocean – Indian Ocean	Global ocean sampling	109,700	118,339,154
Open water	4441611	GS110a – Open Ocean – Indian Ocean	Global ocean sampling	348,823	345,285,679
Open water	4441614	GS110a – Open Ocean – Indian Ocean	Global ocean sampling	110,720	119,426,081
Open water	4441615	GS110a – Open Ocean – Indian Ocean	Global ocean sampling	101,558	105,196,135
Open water	4441616	GS110a – Open Ocean – Indian Ocean	Global ocean sampling	107,966	115,611,614
Open water	4441661	GS023 – Open Ocean – Eastern Tropical Pacific	Global ocean sampling	133,051	143,626,589
Open water	4443740	TA_34838	Sargasso sea bacterioplankton	94,851	16,575,969
Coral reef water	4441121	GS050 – Coral Atoll – Tikehau Lagoon – Fr. Polynesia	Global ocean sampling	715	755,429
Coral reef water	4441133	GS108b – Lagoon Reef – Coccos Keeling, Inside Lagoon – Australia	Global ocean sampling	49,595	53,530,124
Coral reef water	4441139	GS108a – Lagoon Reef Coccos Keeling, Inside Lagoon – Australia	Global ocean sampling	51,788	50,890,568
Coral reef water	4441167	GS048b – Coral reef Moorea, Cooks Bay – Fr. Polynesia	Global ocean sampling	47,692	50,969,448
Coral reef water	4441593	GS025 – Fringing reef – Dirty Rock, Cocos Island – Costa Rica	Global ocean sampling	120,671	129,781,299
Coral reef water	4441603	GS048a – Coral reef – Moorea, Cooks Bay – Fr. Polynesia	Global ocean sampling	90,515	92,813,604
Coral reef water	4441604	GS051 – Coral reef Atoll – Rangirora Atoll – Fr. Polynesia	Global ocean sampling	128,982	140,497,312
Coral reef water	4441617	GS148 – Fringing Reef East coast Zanzibar Tanzania	Global ocean sampling	107,741	107,616,215
Coral reef water	4442642	King14LIMic20070829	Northern line islands	108029	31667620
Coral reef water	4442643	King2LIMic20070817	Northern line islands	97767	37285824
Coral reef water	4442647	Xmas16LIMic20070729	Northern line islands	53169	19900801
Coral reef water	4442648	Xmas29LIMic20070805	Northern line islands	111061	38238805
Coral reef water	4442649	Xmas35LIMic20070808	Northern line islands	44544	15484390
Coral reef water	4442650	Xmas6LIMic20070721	Northern line islands	118943	39280406
Coral reef water	4442651	XmasLag1LIMic20070720	Northern line islands	60531	21801386
Coral reef water	4442652	King7LIMic20070821	Northern line islands	181525	42145245
Coral reef water	4442653	King8LIMic20070823	Northern line islands	119830	37606997
Coral reef water	4440037	KingLIMic20050821	Northern line islands	188,445	19,753,735
Coral reef water	4440039	PalmLIMic20050818	Northern line islands	289,723	30,795,962
Coral reef water	4440041	XmasLIMic20050805	Northern line islands	227,542	23,693,344
Hydrothermal spring	4442583	OCTOPUS	Yellowstone national park	22,272	22,557,192
Hydrothermal spring	4443746	Mushroom springs MatCoreB	Yellowstone national park	2,708	2,713,791
Hydrothermal spring	4443747	Mushroom springs MatCoreD	Yellowstone national park	320	325,932
Hydrothermal spring	4443749	Octopus springs MatCoreF	Yellowstone national park	19,124	18,644,488
Hydrothermal spring	4443750	Octopus springs MatCoreR	Yellowstone national park	1,266	1,328,730
Hydrothermal spring	4443762	Mushroom springs MatCoreF	Yellowstone national park	6,521	6,493,181
Animal associated	4441679	Cow rumen – 640F6	Cow rumen	264,849	26,644,817
Animal associated	4441680	Cow rumen – 80F6	Cow rumen	178,713	18,153,371
Animal associated	4441681	Cow rumen – 710F6	Cow rumen	345,317	35,115,534
Animal associated	4441682	Cow rumen – Pooled Planktonic	Cow rumen	236,830	24,016,021
Animal associated	4440283	Chicken cecum A	Fs-cap	294,682	30,657,259
Animal associated	4440284	Chicken cecum B	Fs-cap	237,940	24,707,007
Animal associated	4440463	Lean mouse cecumMic2005	Human feces – turnbaugh	10,845	8,478,662
Animal associated	4440464	Obese mouse cecumMic2005	Human feces – turnbaugh	11,857	9,067,143
Animal associated	4440056	FishMorGutKentSTMIC20060504	Fish stomach	60,311	5,956,666
